# Motivating smokers at outdoor public smoking hotspots to have a quit attempt with a nicotine replacement therapy sample: study protocol for a randomized controlled trial

**DOI:** 10.1186/s13063-016-1485-z

**Published:** 2016-07-26

**Authors:** Yee Tak Derek Cheung, Jessica Pui Kei Leung, Chelsia Ka Ching Cheung, William Ho Cheung Li, Man Ping Wang, Tai Hing Lam

**Affiliations:** 1 School of Public Health, The University of Hong Kong, Hong Kong, China; 2 School of Nursing, The University of Hong Kong, Hong Kong, China

**Keywords:** Intervention, Nicotine replacement sample, Smoking cessation, Smoking hotspots

## Abstract

**Background:**

About half of the daily smokers in Hong Kong have never tried and have no intention to quit smoking. More than one-third (37.9 %) of daily smokers have attempted to quit but failed. Nicotine replacement therapy (NRT) is a safe and effective pharmacotherapy to increase abstinence by reducing withdrawal symptoms during the early stage of smoking abstinence. However, the prevalence of NRT use in Hong Kong is lower than in most developed countries. The proposed study aims to assess the effectiveness of providing free NRT samples to smokers on increasing quit attempts and the quit rate.

**Methods:**

Trained university undergraduate students as ambassadors will invite smokers at outdoor public smoking hotspots to participate in the randomized controlled trial, in which eligible smokers will be randomized to receive a 1-week free NRT sample and medication counselling (intervention) or advice to purchase NRT on their own (control). The primary outcome is self-reported quit attempts (no smoking for at least 24 hours) in the past 30 days at 1-month and 3-month telephone follow-up.

**Discussion:**

The findings will inform the effectiveness of delivering free NRT samples at outdoor public smoking hotspots to increase quit attempts and abstinence. The study will also provide information on smokers’ adherence to the NRT sample, side effects and safety issues related to the usage. This will improve the design of a large trial to test the effect of the NRT sample.

**Trial registration:**

ClinicalTrials.gov NCT02491086. Registered on 7 July 2015.

**Electronic supplementary material:**

The online version of this article (doi:10.1186/s13063-016-1485-z) contains supplementary material, which is available to authorized users.

## Background

Each year over 5000 deaths are attributable to active smoking in Hong Kong [1]. Quitting smoking is the most effective preventive medicine to reduce the burden of many non-communicable diseases. Hong Kong has the lowest smoking prevalence (10.7 % daily smokers) in the developed world due to the strong tobacco measures enacted in the past few decades [2], but over half (53 %) of daily smokers in Hong Kong have never tried and have no intention to quit smoking [3]. More than one-third (37.9 %) of daily smokers have attempted to quit but failed [3].

Enforcement of the indoor smoking ban led to increased smoking behaviour in outdoor public areas, namely smoking hotspots, where an ashtray is available [4, 5]. These outdoor hotspots were observed in Hong Kong due to the high population density and relatively warm weather throughout the year. Nevertheless, the increased outdoor smoking allows easier identification of smokers and makes way for a face-to-face approach to proactively offer cessation advice outdoors. Since 2009, our research team and the Hong Kong Council on Smoking and Health have organized the ‘Quit to Win’ Contest to promote smoking cessation each year and deliver brief smoking cessation advice for the smokers at these hotspots. This approach recruited 1003 smokers in 3 months to participate in a randomized trial testing the effectiveness of a cash incentive and a brief smoking cessation intervention, and yielded a quit rate of 20 % at 6-month follow-up [6] which warrants development of simple interventions in this setting.

Nicotine is highly addictive and many smokers are unable to quit successfully due to withdrawal symptoms. Nicotine replacement therapy (NRT) is a safe and effective pharmacotherapy to reduce these symptoms during early abstinence and to increase the quit rate in the longer term [7–9]. In Hong Kong, NRT is included in the Hospital Authority Drug Formulary, and can be purchased legally over the counter in a pharmacy or prescribed freely from the Tobacco Control Office’s Quitline and many other smoking cessation clinics. However, the prevalence of using NRT in Hong Kong is lower than in most developed countries. In smokers who had previous quit attempts, 96.8 % tried to quit with ‘self-determination’ and only 23.1 % used prescribed or over-the-counter medication [3]. On the contrary, the corresponding percentage of quitting with medication is at least 40 % in Australia, Canada, the UK, and the USA [10]. The low prevalence of using NRT in Hong Kong may be due to the higher price of NRT per day ($5.43 per day) [11] than, say, in the USA (US$2.41–3.62 per day) [12]. Very few smokers therefore use NRT for their quit attempts, especially those who do not prefer to obtain free NRT from the smoking cessation clinics.

The standard duration of using NRT for smoking cessation is 12 weeks [13, 14]. Providing free NRT for 1 or 2 weeks (i.e. a NRT sample) to motivate smokers to quit is theoretically based on catastrophic theory, suggesting that quitters do not necessarily go through the stages of quitting readiness according to the transtheoretical model [15], but their quit attempts can be initiated by various environmental cues such as sickness [16] and pregnancy [17]. Brief smoking cessation advice and free medication might stimulate smokers to start quitting. Two recent US randomized controlled trials (RCTs) found that a NRT sample increased quitting motivation, confidence and quit attempts in smokers who were not motivated to quit, compared with those who did not receive the sample [18, 19].

The NRT sample might also be beneficial for smokers who have just developed the motivation to quit and will act soon. The Clinical Practice Guideline for smoking cessation recommends that clinicians should advise all smokers to use effective medication for tobacco dependence treatment [13]. Smokers who started to quit shortly after they decided to quit had a higher likelihood of achieving long-term abstinence than smokers who had longer latency between their quitting decision and action [20–22]. Providing a NRT sample and information on NRT for these motivated smokers might be a timely intervention to help them reduce any physical discomfort during quitting and hence increase their abstinence.

Misconception about the safety and efficacy of NRT is associated with a lower interest in using the medication for quit attempts [23–25]. Nearly half of the smokers thought that NRT was as dangerous as cigarettes, and hence underestimated its efficacy to increase quitting success [21]. Providing sufficient and scientific information on NRT is therefore important to enhance the adherence to the NRT. We also suggest that increasing literacy in medication, and enhancing smokers’ experience in using cessation aids are beneficial. Because both abrupt and gradual cessation with NRT are effective to increase abstinence for motivated and unmotivated smokers [26, 27], NRT users should be well informed about these approaches while making their own decisions.

We propose to conduct a RCT to evaluate the effectiveness of a NRT sample in adult smokers recruited at smoking hotspots. The setting for the recruitment and intervention of the proposed RCT will be similar to the aforementioned Quit to Win Contest, but we will use a free NRT sample as the major intervention instead of a cash incentive. The primary research questions of the present RCT are: will a NRT sample increase quit attempts and abstinence in smokers who are smoking at smoking hotspots; and will a NRT sample increase motivation to quit?

## Methods

### Trial design

**Fig. 1:**
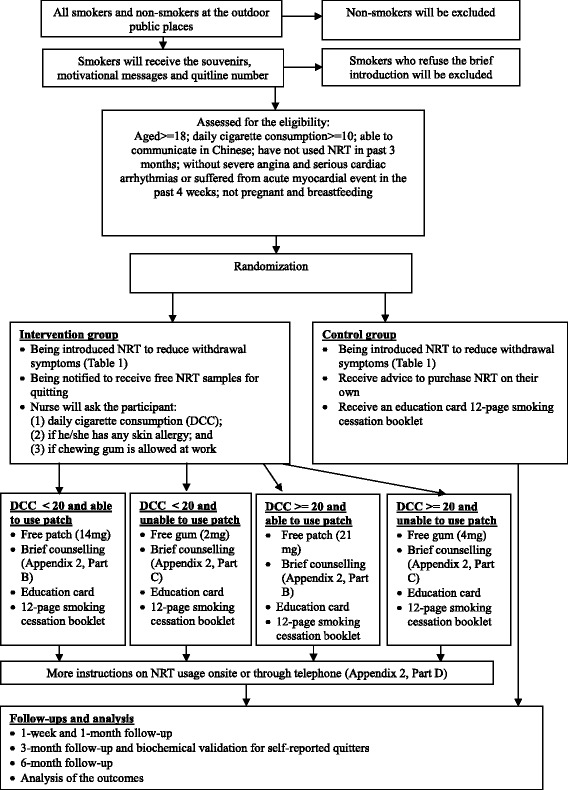
Flow of outreach interventions. *NRT* nicotine replacement therapy

The study aims to motivate smokers to have a quit attempt with a free NRT sample. We will firstly train nurses and university undergraduate students as outreach smoking cessation ambassadors, who will proactively approach the smokers at outdoor smoking hotspots of urban areas, where rubbish bins with a collector of cigarette butts are nearby, and invite them to participate in the two-arm, parallel-group RCT. The subject recruitment will take place at nine selected outdoor smoking hotspots where many smokers will remain and smoke from July 2015 to January 2016. Informed consent will be obtained from all participants. The study flow and the schedule of the study procedures are indicated in Fig. 1 and Table 1, respectively. Our trial design has followed the SPIRIT checklist for standard protocol items (see Additional file 1).

**Table 1:** Schedule of enrolment, interventions and assessments

	Study period					
	Enrolment	Allocation	Post-allocation			
Time point	Baseline	Baseline	1 week	1 month	3 months	6 months
Enrolment						
Eligibility screen	X					
Informed consent	X					
Allocation		X				
Interventions						
Intervention group		X				
Control group		X				
Assessments						
Socio-demographic	X	X				
Daily cigarette consumption		X	X	X	X	X
Intention to quit		X	X	X	X	X
Self-reported quitting outcomes			X	X	X	X
Perceived importance, confidence and difficulty to quit			X	X	X	X
Use of NRT			X	X	X	X
Biochemically validated quitting outcomes			X	X	X	X

*NRT* nicotine replacement therapy

### Subjects

Smokers with the following inclusion criteria will be invited to participate in our RCT: aged 18 years or older; smoked 10 cigarettes or more per day in the past week; able to read and speak Chinese; have not used NRT for the past 3 months; have no severe angina and serious cardiac arrhythmias; have not suffered an acute myocardial event in the past 4 weeks; and not pregnant and breastfeeding.

### Procedures

We will train the smoking cessation ambassadors for the subject recruitment and delivery of interventions to the subjects. Recruitment of outreach smoking cessation ambassadors will be conducted through sending mass mails to students of the University of Hong Kong. Retired nurses who are now providing counselling services in our existing smoking cessation projects will also be recruited. They will be invited to participate in a training programme about smoking cessation counselling and pharmacotherapy in assisting smokers to quit smoking. Our research team (comprised of physicians and nurses) will develop the training materials and intervention guides based on the principles of the US Agency for Health Care Policy and Research (AHCPR) guidelines [28] and the guidelines of the National Institutes of Health [29] and World Health Organization [30]. The training content will cover: the tobacco epidemic in Hong Kong; the health hazards of smoking; basic skills in assessing smoking dependence and quitting readiness; knowledge of smoking cessation medication (types of NRT, adherence, side effects, etc.); and brief counselling skills. At the end of the training, participants should be capable of delivering a brief smoking cessation intervention, which includes providing advice on using NRT.

Smoking cessation ambassadors will be paired up to approach the smokers and distribute souvenirs at the outdoor smoking hotspots in daytime. The souvenirs will include a small pack of tissue paper, with motivational messages (e.g. one in two smokers will be killed by smoking), quitline number (e.g. Quitline of Tobacco Control Office 1833183) and other resources for smoking cessation services printed on the tissue-paper pack. If the smoker is willing to accept the souvenir and talk to the ambassador, one ambassador will ask further questions related to eligibility through an informal conversation. If the subject is eligible, the ambassador will introduce our RCT and NRT (about its side effects), seek his/her consent to participate and complete a one-page questionnaire on his/her behalf. The other ambassador will then randomize the subject by the sequentially numbered, opaque sealed envelopes (SNOSE) method.

### Interventions

The ambassador will help the subjects in the intervention group decide which type of NRT product (patch or gum) he/she can use and advise him/her on how to use the NRT based on his/her smoking habit and daily cigarette consumption. He/she will also be provided with an education card about NRT and a 12-page smoking cessation booklet (Fig. 1). Based on the experience of the previous trials [11, 31, 32], the choice of NRT (patch or gum) will be made according to the subject’s preference and the ambassador will provide medication counselling. Afterwards, the subjects will receive a free pack of 1-week NRT. If the subject is willing to continue the counselling at recruitment, the ambassador will introduce the NRT’s side effects, adherence and effectiveness (Table 2). Otherwise, the ambassador will contact the subject to provide such details and enquire about his/her usage of NRT by telephone within 2 days.

**Table 2:** Counselling content for the intervention group

Part A: Introduction of NRT
1. Quitting smoking may cause withdrawal symptoms, such as irritability, insomnia, frustration, anxiety, restlessness and craving for cigarettes
2. Nicotine gum and patch can help relieve these symptoms
3. Nicotine products are far less dangerous than cigarette smoking
4. Scientific evidence strongly supports that NRT increases the quit rate and is a safe product.
Part B: Brief instruction for NRT patch
1. Apply patch on clean and dry skin on the chest, back, upper arms, hips, etc.
2. Apply one patch per day and remove before sleep
3. Change the patch site daily to avoid skin irritation
Part C: Brief instruction for NRT gum
1. Steps of chewing gum: slowly chew the gum 10–15 times → the taste gradually becomes stronger → park the gum in the buccal area for about 1–2 minutes → the taste gradually becomes lighter → repeat the above steps
2. Avoid soft drinks, coffee and fruit juice 15 minutes before chewing
3. Daily dosage should not be more than 15 pieces
Part D: Detailed instructions on NRT usage
1. Minor side effects may appear such as insomnia, skin irritation, jaw ache, hiccups and mouth soreness, but they will disappear after a few days
2. Make using NRT a daily habit
3. The standard medication period is 8 weeks. Suggest the participants continue medication after using all samples
4. Suggest the participants continue to use patch/gum even though feeling able to maintain abstinence
5. Must reduce or quit smoking during using patch/gum, otherwise it will increase the intake of nicotine
6. Tell others that you are using NRT, so that they can remind you to use it
7. Advise the participant to have healthy food intake and exercise to prevent weight gain during quitting
8. Our counsellor will call the participants to follow-up the usage after 1 week
9. Ask the participant to send back unused NRT to us in the enclosed envelope
10. Call the quitline (1833183) for further enquires

*NRT* nicotine replacement therapy

The control group subjects will be given the same advice on using NRT by the ambassador as the intervention group. The subjects will be suggested to purchase NRT on their own, but will not be given the sample. The same education card and the smoking cessation booklet will be provided.

### Outcomes

The primary outcome is the proportion of any self-reported quit attempts (no smoking for at least 24 hours) in the past month at 1-month and 3-month follow-up. The secondary outcomes are self-reported 7-day point prevalence of abstinence at 1-month, 3-month and 6-month follow-up, perceived importance, confidence and difficulty to quit (scale 0–10) at all follow-up stages, proportion of using NRT in the past week or past month, and biochemically validated abstinence at 1-month follow-up.

At 1 week and 1, 3 and 6 months after recruitment, all subjects will be contacted by a trained interviewer via telephone for a survey of assessing these outcomes. After the survey, the interviewer will discuss any difficulties in quitting and using NRT with the subject. At least seven call attempts at different time points will be made before the subject is considered a loss to follow-up. Subjects who report no smoking in the past 7 days will be considered self-reported quitters. Self-reported quitters at 1 month will be invited to participate in a biochemical validation including measurement of exhaled carbon monoxide (CO) and salivary cotinine level by NicAlert® strips (http://www.nymox.com/default.action?itemid=45) near their residence. The criteria for validated abstinence were exhaled CO < 4 ppm and salivary cotinine < 10 ng/ml [33, 34].

### Sample size determination

Since the RCT will generate preliminary estimates for effectiveness of the NRT sample, we propose the sample size as 50 for each arm (i.e. total sample size = 100). A similar RCT conducted by Jardin et al. [19] which evaluated the effectiveness of 2-week free NRT to help non-motivated smokers to quit smoking was used for the power estimation. The results showed that the 3-month quit attempt rate of receiving free NRT was significantly higher than for those who only received a quitline referral (32 % vs 16 %, *p* = 0.05). The relative risk (RR) is therefore determined (32 %/16 % = 2.0). Based on the RR of 2.0 and the 100 subjects, the power and type I error for detecting the difference using the Fisher’s exact test will be 69.7 % and 0.08. Based on our recent experience with the same recruitment method and setting, the successful recruitment rate was about 15.4 % (1254/8063). We therefore estimate we have to approach 650 smokers to achieve the target sample size.

### Randomization

The individual randomization method by SNOSE will be used to ensure the allocation sequence is concealed from both ambassadors and participants before the group allocation [35, 36]. The primary investigator will prepare about 150 identical, opaque, sealed, A5-sized envelops, with a unique three-digit serial number on the cover of each envelope as an identifier. Half of the envelopes will each contain an eligibility form, an education card of NRT and an action plan for the intervention group. The remaining half will contain the same eligibility form and education card, and an action plan for the control group. After inserting the intervention materials in the envelopes, they will be shuffled and then numbered. When the subject consents to participate, the ambassadors will open one envelope according to the sequence of the serial number and assign the treatment condition based on the action plan.

### Allocation concealment

The group allocation will not be known by the subjects and the ambassador before the assignment allocation. In reality, smokers often linger and smoke for just about a few minutes at the hotspots, and then leave. Also, there are far more smokers than ambassadors in our selected outdoor hotpots. Therefore, the smokers may not notice the procedures of the enrolment of other subjects clearly. In addition, not all smokers want to be approached by strangers, and finally very few of them will agree to participate in the RCT, which is expected. Therefore, our ambassadors can rarely approach and recruit a new subject quickly after finishing the enrolment of a previous subject. It is unlikely that their decision to join the RCT is greatly influenced by what they have observed from others’ enrolment and intervention. Although the control group subjects may see that other subjects receive the NRT sample, the impact on the enrolment and group allocation is limited in reality.

### Blinding

Outcome assessors at follow-up will be blinded to the treatment condition of each participant when they assess the primary and secondary outcomes, but they may not be blinded in future follow-up due to disclosure of the treatment condition by the subject during the interview*.* To prevent this, follow-up duties will be done by five or more interviewers, and follow-up of a particular subject may be done by different interviewers. The biochemical validation will be done by a staff member who has not delivered the intervention and conducted the telephone follow-up. Yet all subjects will not be blinded, because they will receive the behavioural intervention.

### Statistical analyses

All data will be entered and analysed by SPSS for Windows version 20. The rate of quit attempts and 7-day point prevalence of abstinence will be assessed with the chi-square test and odds ratios of logistic regression. Continuous variables of perceived importance, confidence and difficulty to quit will be tested with repeated-measures ANOVA. Both intention-to-treat (assuming missing subjects have no changes) and complete case analyses will be done.

### Data quality control

Either the research nurse, the principal investigator or the research coordinator will coordinate the fieldwork at each recruitment site. All of the fieldwork and data management will be monitored by the other two co-investigators (MPW and WHCL). The principal investigator will randomly select five intervention sites by simple individual randomization for checking of intervention fidelity. The research assistant and the investigators will have weekly meetings to review the study progress and procedures and to discuss any adverse events or dropouts. In view of the small sample size and short study period, a data monitoring committee, interim analysis and stopping guidelines will not be necessary. The data collection, management, analysis, interpretation and production of publications will be independent from the funding bodies and other competing interests. The trial results will be disseminated via journal publication and conference presentation, without exposing the identity of the trial subjects.

### Ethics, consent and permissions

The study protocol has been approved by the Institutional Review Board of the University of Hong Kong/Hospital Authority Hong Kong West Cluster (IRB reference number: UW 15-232). Written consent will be sought from all subjects to participate in this RCT, which will permit the investigators to disseminate the trial results and the study protocol via publications, without showing individual information.

All questionnaires will be stored in a cupboard with keys kept by the principal investigator and the research assistant only. The data will be kept for 10 years upon completion of the study. Electronic datasets of personal information and contact information will be encrypted and separated from the research dataset during the study period, and will be destroyed after the said storage period.

NRT has been proven an effective and safe aid for smoking cessation, which can be purchased over the counter in pharmacies or prescribed freely in smoking cessation clinics in Hong Kong. A common side effect of a nicotine patch is skin reaction. About 50 % of patients using the nicotine patch will experience a local skin reaction. The reaction is usually mild and self-limiting, but occasionally worsens over the course of therapy. Local treatment with hydrocortisone cream (1 %) or triamcinolone cream (0.5 %) and rotating patch sites may ameliorate such local reaction. In fewer than 5 % of patients, such reaction requires the discontinuation of the NRT. Other side effects are insomnia and/or vivid dreams. Common side effects of nicotine gum include mouth soreness, hiccups, dyspepsia and jaw ache. These effects are generally mild and transient, and can often be alleviated by correcting the patient’s chewing technique [13] or correcting the patient’s using habit. All subjects will be asked at telephone follow-up if they suffer from these side effects. If so, the counsellor will provide counselling on the usage. If the side effects are serious, the counsellor will ask them to cease usage.

## Discussion

Previous studies strongly supported that NRT is a safe and effective smoking cessation aid for smokers [7–9], but the low literacy reduces the using prevalence. Despite the widespread promotion of smoking cessation messages, an increase in the proportion of hardcore smokers and a reduction in the quit rate support that more effective methods for promoting cessation aids are needed [37]. The present RCT will assess whether providing a NRT sample is an effective health promotion strategy to enhance more quit attempts and increase abstinence. In addition to the effectiveness in quitting, the present RCT will yield more information on the adherence to the NRT sample, side effects and safety of NRT usage, and will also test whether the NRT sample and counselling increase quit motivation. These findings will provide insights towards enhancing smoking cessation services and constructing a larger trial to test the population effect in the near future.

A proactive approach with telephone ‘cold-calling’ or health records has been used by other studies, and was effective to increase abstinence [38, 39]. The present study is the first to extend this approach by directly initiating a face-to-face conversation with the smokers and then providing medication samples at smoking hotspots. These smokers may be less motivated to quit than those who seek cessation services through clinics or quitline. Because unmotivated smokers comprise a large proportion of smokers in Chinese communities, both in the China mainland and Hong Kong [3, 40], the present study will increase understanding on how to help these unmotivated smokers quit.

## Trial status

The recruitment is ongoing.

## Abbreviations

NRT, nicotine replacement therapy; RCT, randomized controlled trial; RR, relative risk; SNOSE, sequentially numbered, opaque sealed envelopes

## Additional file

Additional file 1: SPIRIT checklist for standard protocol items. (DOC 117 kb)
